# Deep Brain Stimulation of the Subthalamic Nucleus Improves Lexical Switching in Parkinsons Disease Patients

**DOI:** 10.1371/journal.pone.0161404

**Published:** 2016-08-30

**Authors:** Isabelle Vonberg, Felicitas Ehlen, Ortwin Fromm, Andrea A. Kühn, Fabian Klostermann

**Affiliations:** 1 Dept. of Neurology, Motor and Cognition Group, Charité–University Medicine Berlin, Berlin, Germany; 2 Dept. of Neurology, Motor Neuroscience Group, Berlin, Charité–University Medicine Berlin, Germany; University of Medicine & Dentistry of NJ - New Jersey Medical School, UNITED STATES

## Abstract

**Objective:**

Reduced verbal fluency (VF) has been reported in patients with Parkinson’s disease (PD), especially those treated by Deep Brain Stimulation of the subthalamic nucleus (STN DBS). To delineate the nature of this dysfunction we aimed at identifying the particular VF-related operations modified by STN DBS.

**Method:**

Eleven PD patients performed VF tasks in their STN DBS ON and OFF condition. To differentiate VF-components modulated by the stimulation, a temporal cluster analysis was performed, separating production spurts (i.e., ‘clusters’ as correlates of automatic activation spread within lexical fields) from slower cluster transitions (i.e., ‘switches’ reflecting set-shifting towards new lexical fields). The results were compared to those of eleven healthy control subjects.

**Results:**

PD patients produced significantly more switches accompanied by shorter switch times in the STN DBS ON compared to the STN DBS OFF condition. The number of clusters and time intervals between words within clusters were not affected by the treatment state. Although switch behavior in patients with DBS ON improved, their task performance was still lower compared to that of healthy controls.

**Discussion:**

Beyond impacting on motor symptoms, STN DBS seems to influence the dynamics of cognitive procedures. Specifically, the results are in line with basal ganglia roles for cognitive switching, in the particular case of VF, from prevailing lexical concepts to new ones.

## Introduction

Reduced performance in verbal fluency (VF) tasks is a robust finding in patients with Parkinson’s disease (PD), in particular if treated by Deep Brain Stimulation of the subthalamic nucleus (STN DBS) [1–4] (for reviews see [5,6]). The nature of this abnormality and its relation to the basal ganglia (BG) dysfunction in PD are unclear. Comparisons between VF performance in PD patients before versus after brain surgery for STN DBS consistently hint at a negative impact of DBS surgery on word production [7–12], but the effect of the STN DBS itself, as assessed by investigations under active versus inactive stimulation (i.e., ON and OFF conditions), remains vague [13,14]. Reasons for this could be that stimulation effects on VF performance are much weaker than surgery-related changes, on which they are superimposed, and that the functional state of the BG is only relevant for particular aspects of word production.

Principally, the BG are presumed to play an important role for balancing the release of competing cortical input, thereby controlling the maintenance versus exchange of ongoing actions [15]. Tying in with this notion, the inability to flexibly switch between different elements as well as between types of motor and non-motor behaviors in PD is considered as a characteristic sequel of BG dysfunction [15–17]. Improvement of frontostriatal signaling by PD treatment, be it STN DBS or dopaminergic therapy, seems to ameliorate respective deficits [18–21], e.g., facilitating set-shifting operations for mental strategies in Trail Making and Stroop tests [21,22].

In the current context it is important to note that the search process for VF tasks is thought to comprise two major aspects [23,24], (i) the recruitment of word-related information belonging to a common semantic concept, and (ii) transitions to other semantic fields whenever the search process within a field is exhausted, i.e. no further lexical items are available in the field. From a network perspective, the first process is thought to rely on rapid automatic activation spread over densely interconnected neuronal ‘association nodes’ [24–26], whereas the second one is categorized as an attention-demanding, slower set-shifting operation. Support for this concept comes from ‘temporal cluster and switching analysis’ [23], a mathematical procedure for the formal assessments of VF output dynamics. Based on this approach, it could be demonstrated that words produced in ‘clusters’, i.e., intervals with rapid verbal production, are more semantically related than words with longer pauses in between, representing ‘switches’ [23,27].

To study whether and how different functional BG states impact on lexical switching as a minimal form of mental set-shifting, we subjected the VF performances of patients in ON versus OFF STN DBS conditions to temporal cluster analyses. Based on the above, we presumed that the disengagement from a prevailing lexical concept towards another one is a particular problem in PD, resulting in reduced VF. We therefore hypothesized that STN DBS as an approach for the restoration of impaired BG function would facilitate deficient switching rather than impacting on lexical cluster-related processes of word production. The patients’ results were compared to those of healthy controls and are discussed in the framework of PD treatment actions on cognitive corticobasal function.

## Materials and Methods

Eleven patients with PD and bilateral DBS of the STN were recruited for this study from the Outpatient Clinic for Movement Disorders of the Charité Berlin. All met the United Kingdom Brain Bank Criteria for PD. Exclusion criteria were: (i) dementia (cut-off value < 14 points in the Parkinson Neuropsychometric Dementia Assessment, PANDA [28]) and (ii) brain diseases other than PD, including depression (based on the criteria of the German Manual for Psychopathological Diagnosis, AMDP [29]). All patients were on stable levodopa medication, mostly combined with other, mainly dopaminergic antiparkinsonian medication. The total daily levodopa equivalence dose (LED) was determined according to recommended conversion factors [30]. Eleven age and education-matched healthy subjects formed a control group. All participants were native German speakers and gave their written informed consent to the study protocol, approved by the Ethics Committee of the Charité in accordance with The Code of Ethics of the World Medical Association (Declaration of Helsinki).

Patients were studied under continued as well as paused DBS treatment (ON and OFF stimulation conditions). The order of tests (ON first versus OFF first) was counterbalanced. The interval between ON- and OFF-sessions was two months. The OFF treatment condition was defined as a stimulation pause of at least 30 minutes before the test session under inactivated DBS was started. In both the ON and the OFF condition, patients continued to receive their regular medication.

The positions of active DBS electrodes were derived from post-operative MRI. Coordinates are expressed as points normalized in the standard Montreal Neurological Institute (MNI) stereotactic space [31]. MNI-localizations were determined for the geometrical center of the MRI susceptibility artefact of each active electrode contact. The x, y and z data specify localizations on the defined medio-lateral, antero-posterior, rostro-caudal MNI axis per hemisphere with (with a reference point close to, but not exactly identical with the midpoint of the AC-PC line). For the active electrodes atlas-specific coordinates were calculated per hemisphere. The total electrical energy delivered (TEED _1sec_) was computed as [32]:

$$\left(\frac{{voltage}^{2}*pulsewidth*frequency}{impedance}\right)*1\;sec.$$

Subjects‘ characteristics and DBS parameters are summarized in Table 1 and Table 2.

**Table 1:** Baseline Characteristics.

	Controls	Patients ON condition	
	(n = 11)	(n = 11)	
	Mean ± SD	Mean ± SD	*p*-values
	(Range)	(Range)	(if applicable)
**Age (years)**	64.91 ± 5.86	64.64 ± 8.90	.93
	(52–71)	(48–77)	
**Education (years)**	10.73 ± 1.90	10.45 ± 1.63	.72
	(8–13)	(8–13)	
**Gender (f/m)**	3 / 8	2 / 9	.61
**Handedness (r/l)**	8 / 3	10 / 1	.27
**net PANDA (points)**	18.64 ± 3.07	17.18 ± 3.68	.33
	(13–23)	(13–23)	
**Disease Duration (years)**		13.55 ± 5.48	
		(4–22)	
**LED (mg)**		520.05 ± 454.29	
		(0–1300)	
**UPDRS-III (points)**		19.64 ± 8.59	
		(11–40)	
**HY (stage)**		2.55 ± 0.69	
		(2–4)	
**DBS Duration (years)**		3.36 ± 2.37	
		(0.5–7)	

Overview of patients in their ON stimulation condition and control subjects (Contr.). net PANDA: Parkinson Neuropsychometric Dementia Assessment (PANDA) score without VF test items–maximum 23 points; LED: levodopa equivalent dose per day; duration: disease duration; UPDRS-III: Unified Parkinson’s Disease Rating Scale–motor score (maximum 108 points); HY: Hoehn & Yahr score.

**Table 2:** Stimulation Parameters.

	right	left
	Mean ± SD	Mean ± SD
**Amplitude (V)**	2.93 ± 1.61	3.11 ± 1.39
**Pulse width (μs)**	62.73 ± 9.05	65.45 ± 12.14
**Frequency (Hz)**	135.45 ± 30.78	135.45 ± 30.78
**TEED**	121.61 ± 117.00	152.32 ± 144.58
**Polarity (mono / bi)**	10 / 1	10 / 1
**Position of center of active contacts**		
**x (mm)**	11.79 ± 0.76	12.02 ± 1.12
**y (mm)**	-14.32 ± 1.10	-13.89 ± 1.10
**z (mm)**	-6.58 ± 1.43	-6.73 ± 1.69

Shown are the mean stimulation parameters for the left and right hemisphere. Electrode positions correspond to the positions of the active electrodes in the standard Montreal Neurological Institute (MNI) stereotactic space along the medio-lateral (x), antero-posterior (y), rostro-caudal (z) MNI axis per hemisphere. Values indicate the mean (± standard deviation).

### Procedure

All participants performed a standard German VF test (‘Regensburger Wortfluessigkeitstest’ [33]) demanding to name as many words as possible within two minutes under four task conditions: two semantic tasks (naming words from the category ‘vegetables’, and from the categories ‘animals’ and ‘furniture’ alternatingly), and two phonemic tasks (naming words starting with ‘s’, and naming words starting with ‘g’ and ‘r’ alternatingly). Metacomments (e.g., ‘I don’t know any more words’) were excluded from the analysis. In accordance with previous studies in this field, not allowed repetitions, words with the same word stem, and proper names were left in the analysis since they are generally considered to be informative about the underlying search processes [34].

The order of the tasks was randomized for each patient. The subjects’ responses were digitally recorded (Audacity® version 1.3.13-beta).

### Cluster and Switching Analysis

The VF output was analyzed with Audacity^®^ (analysis software for digitally recorded language production; operating system: Windows^®^). Word durations and pause lengths between words were measured at a temporal resolution of 1ms.

**Fig 1:**
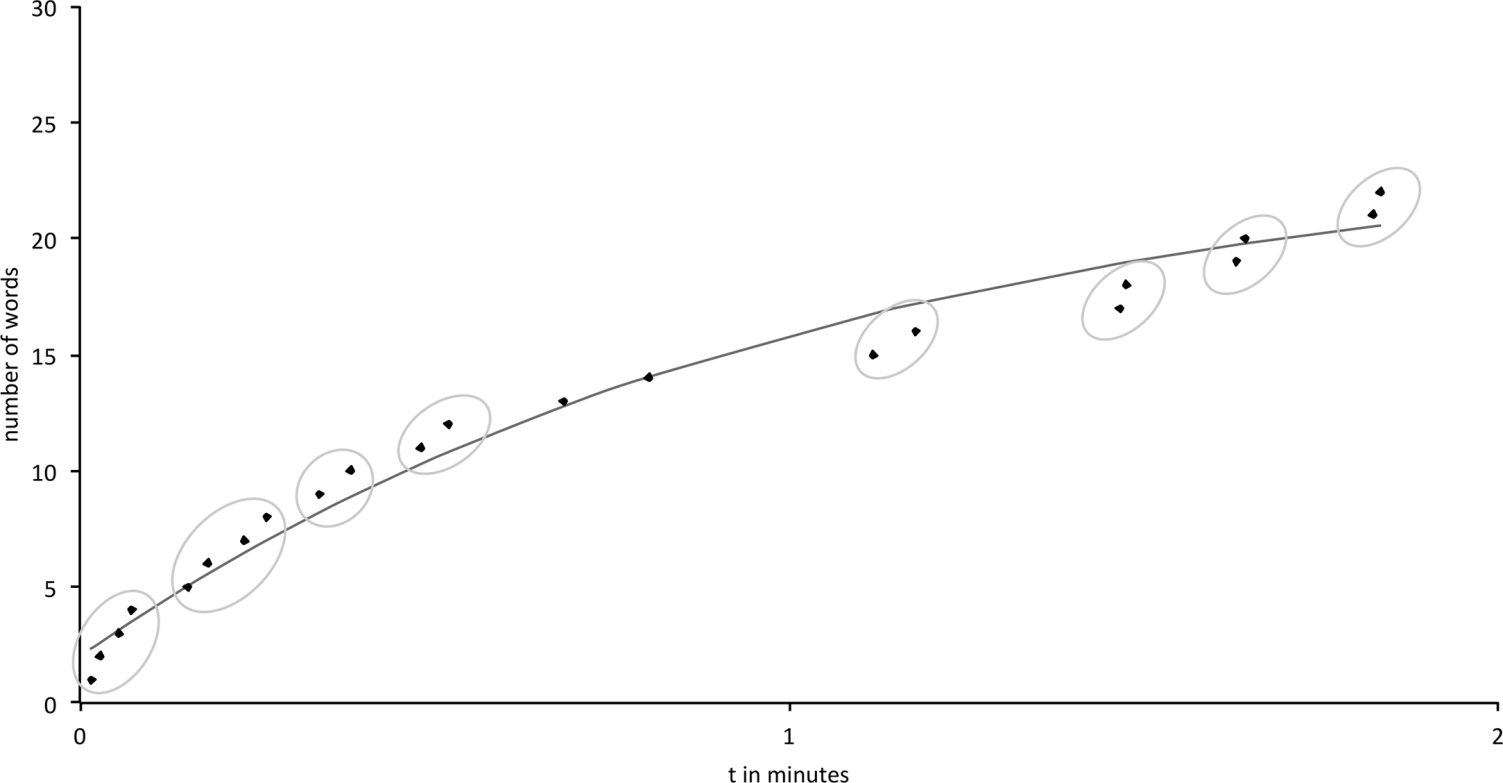
Example of a curve fit. Scheme of best-fitting curve for an individual VF course; circled segments represent clusters with faster than predicted word production as opposed to switches between thus defined clusters.

In order to perform a temporal cluster and switching analysis, curve fittings of the individual word production times were performed using the exponential function *n*(*t*) = *c* * (1 − *e*^−*mt*^) [35]. This formula has specifically been developed and used for describing the progression of cumulative word production in corresponding tasks and provides a reliable basis for further cluster analysis (for a review see [36]). Its graph starts at the origin and approaches the asymptote *c*, with *m* indicating the rate of growth to the asymptote and *n(t)* the number of produced words. To obtain the best curve fitting, the function can be linearized by taking the logarithm of the exponential function in order to subject the variation of the results to a least-mean-square analysis. In so doing, the optimal exponential function for the individual verbal output dynamics was identified. For each individual curve, clusters and switches were afterwards determined using the slope-difference algorithm proposed by Gruenewald & Lockhead [23]. Accordingly, words were defined as belonging to one cluster, if the slope between them was steeper than the local slope predicted by the best-fitting exponential function. Switches were defined as shifts between thus identified clusters, i.e. when the slope between two following words was lower than the predicted one [37]. For an example of a curve fit see Fig 1.

The following parameters were examined for each participant in each task condition: (i) the total *number of words* produced, (ii) total *number of clusters*, (iii) *intra-cluster time* (the interval between consecutive words within the same cluster), (iv) total *number of switches*, and (v) *switch time* (the interval between consecutive words belonging to different clusters).

### Statistical analysis

#### Test of the Hypothesis

To identify effects of the STN DBS treatment on the *number of switches*, we performed an analysis of variance (ANOVA) containing three within-subject factors, i.e., ‘*treatment state*’, ‘*task condition alternation*’, and ‘*task condition phonemic vs*. *semantic*’ (each with two levels).

#### Additional Evaluation

Further ON-OFF ANOVAs were run for the *number of words*, *number of clusters*, *intra-cluster times*, and *switch times*. To additionally compare the task performance between patients in the DBS ON condition as well as in the DBS OFF condition with that of the control group, further ANOVAs were carried out for the *number of switches*, *number of clusters*, *intra-cluster times*, *number of words*, and *switch times* with the between-subject factor ‘group’ (two levels) and the within-subject factors ‘*task condition alternation*’, and ‘*task condition phonemic vs*. *semantic*’.

Significant differences were assumed at a Bonferroni-corrected *p* ≤ .05.

Pearson’s correlations for normally distributed data were calculated between the significant ON-OFF-related changes with the UPDRS and with stimulation parameters (*amplitude*, *frequency*, *pulse width*, *TEED*_*1sec*_ and *localization of active contacts*).

The statistical analyses were performed with SPSS^®^ version 19.

## Results

### Clinical and subject-related characteristic*s*

PD patients and controls did not differ significantly with respect to *education*, *gender*, *handedness*, or *age* (see Table 1).

*The motor UPDRS* of DBS patients was significantly improved by the STN stimulation (ON: 19.64 ± 8.59; OFF: 38.45 ± 15.86; *p* < .001).

### VF performance

**Fig 2:**
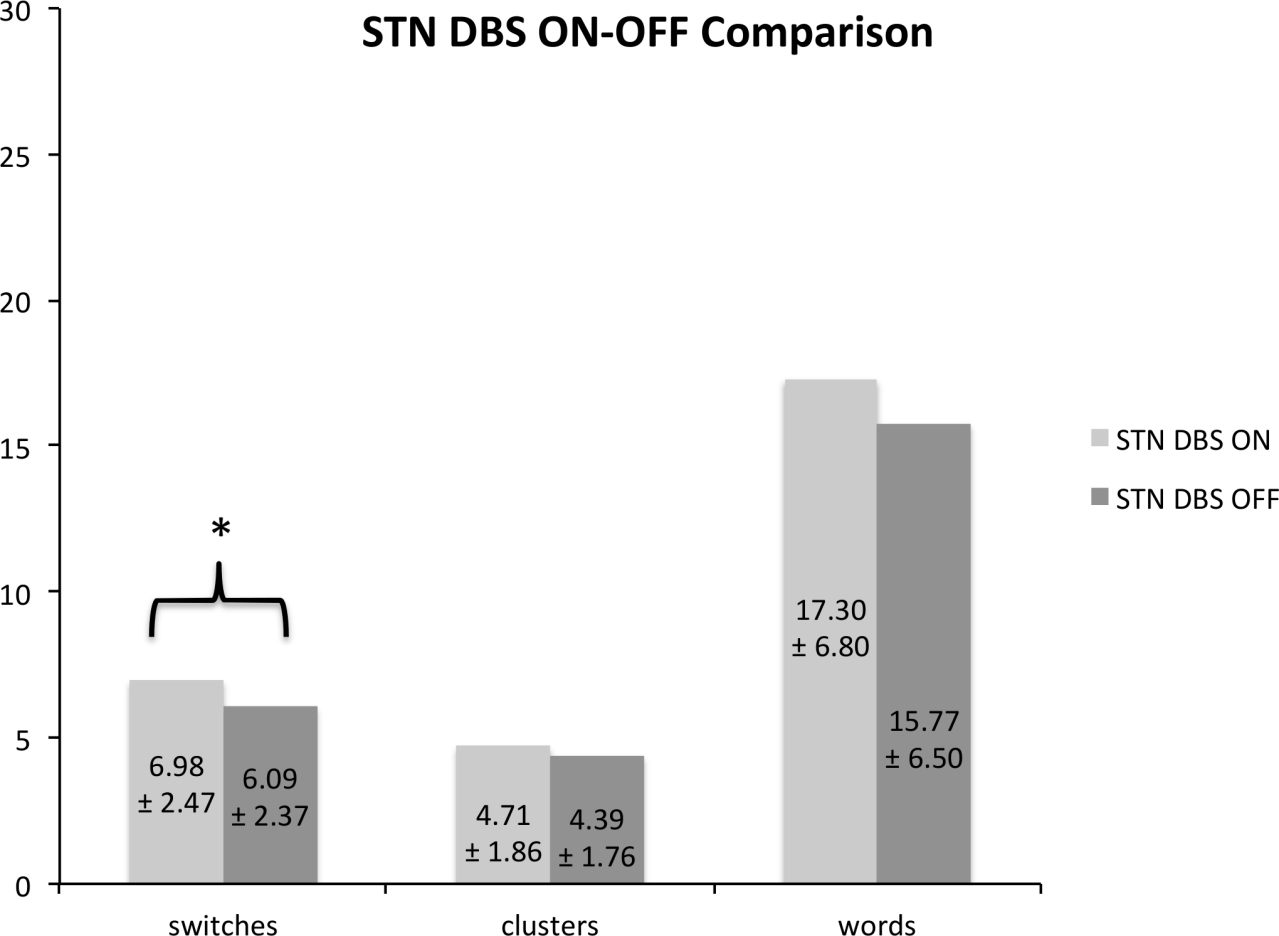
STN DBS ON-OFF Comparison. Fig 2 shows the ON-OFF comparison of VF performance in the patient group. Values indicate the mean (± standard deviations) for the number of switches, the number of clusters, and the number of words. * significant treatment-related effect at a *p*-level < .05.

Fig 2 provides an overview over the mean values of the *number of switches*, *number of clusters*, and *number of words* of the patients’ ON-OFF comparison.

### Test of the Hypothesis

The ON-OFF ANOVA showed a significantly higher *number of switches* in the DBS ON compared to DBS OFF condition (ON: 6.98 ± 2.47; OFF: 6.09 ± 2.37; *F*_1,10_ = 6.380; *p* = .030).

### Additional Evaluation

Compared to the control group, the *number of switches* was significantly lower in PD patients regardless of the stimulation condition (controls: 9.82 ± 3.04, patients in ON / OFF: 6.98 ± 2.47 / 6.09 ± 2.37; *F*_1,20_ = 11.071, *p* = .003 / *F*_1,20_ = 18.71, *p* < .000).

The *number of clusters* did not differ significantly between the DBS ON and OFF condition (ON: 4.71 ± 1.86, OFF: 4.39 ± 1.76; *F*_1,10_ = 1.156; *p* = .308). However, compared to healthy controls, it was significantly lower in patients, both in the ON and OFF stimulation condition (controls: 6.32 ± 2.10, patients in ON / OFF: 4.71 ± 1.86 / 4.39 ± 1.76; *F*_1,20_ = 7.850, *p* = .011 / *F*_1,20_ = 9.344, *p* = .006).

With respect to the *number of words*, no ON-OFF effect was found (ON: 17.30 ± 6.80, OFF: 15.77 ± 6.50; *F*_1,10_ = 9.389; *p* = .127), but generally PD patients generated significantly fewer words than controls (controls: 25.10 ± 7.98, patients ON / OFF: 17.30 ± 6.80 / 15.77 ± 6.50; *F*_1,20_ = 9.389; *p* = .006 / *F*_1,20_ = 14.431, *p* = .001).

Regarding the *switch times*, a trend towards an ON-OFF effect was found (ON: 13.85 ± 13.77, OFF: 16.65 ± 16.20; *F*_1,10_ = 3.820; *p* = .079). Compared to controls patients in both stimulation conditions had significantly longer switch times (controls: 7.61 ± 3.10, patients ON / OFF: 13.85 ± 13.77 / 16.65 ± 16.20; *F*_1,20_ = 8.074, *p* = .010 / *F*_1,20_ = 9.855, *p* = .005).

The *intra-cluster times* were not significantly different in the DBS ON versus OFF condition (ON: 3.85 ± 3.99, OFF: 4.33 ± 2.12; *F*_1,10_ = .917; *p* = .361), but PD patients had significantly longer intra-cluster times compared to controls (controls: 2.16 ± 0.99, patients ON / OFF: 3.85 ± 3.99 / 4.33 ± 2.12; *F*_1,20_ = 5.291, *p* = .032 / *F*_1,20_ = 14.922, *p* = .001).

### Correlations

No significant correlation was found between the change score of the *number of switches* and the *UPDRS motor score* (*r* = .035; *p* = .919). A positive correlation between the increase in *the number of switches* and the TEED_1sec_ in the left hemisphere was found (*r* = .724; *p* = .012).

## Discussion

In this study we performed a temporal cluster and switching analysis for the VF performance of PD patients with STN DBS in ON versus OFF stimulation conditions. In so doing, potential effects of functional BG modulations on lexical *switching*–as a procedural element underlying word production–were assessed. In sum, DBS went along with a higher number of switches alongside with a trend towards reduced switch time. Changes of the respective cluster parameters were not found. The comparisons with healthy control subjects revealed that the patient group generally performed abnormal, and that their VF performance was not compensated by the subtle stimulation-related changes observed. Even in the better ON condition the values for clusters, word numbers, switches and intra-cluster times as well as switch times remained below normal levels.

Altogether, the stimulation-related improvement of switching functions is reminiscent of effects that STN DBS unfolds on motor symptoms in PD patients. It might counteract the maintenance of ‘static’ lexical concepts during VF task performance and, in so doing, finally support mental flexibility.

### Conceptual considerations

Generally, switches in VF are supposed to refer to frontal executive functions, whereas clusters reflect lexico-semantic processes [34]. The switch-increase and the concomitant slight reduction in switch times in the ON-condition therefore suggest that STN DBS rather impacts on the procedural than on the lexical aspect of VF performance. Indeed, an involvement of the STN in lexical switching has been proposed by neurophysiological recordings from STN DBS electrodes in PD patients performing VF tasks [38]. Specifically, the observation of increased gamma-band activity during semantic switching after STN DBS surgery has led to the assumption that perturbation of switch-related STN functions could account for VF impairments [38] often reported in DBS patients post-operatively [39–41].

However, concerning the neuromodulation of STN function, significant differences of overall VF performance were not reported when comparing patients’ in ON vs. OFF DBS conditions [42–44]. Instead, DBS surgery in PD patients was found to be associated with a marked decline of word production in respective tasks, e.g., thought to result from a disruption of fronto-basal connections alongside the electrode trajectory to the STN [9,12,45]. Thus, although the neurophysiological findings by Anzak et al. [38] probably indicate some involvement of the STN in the mediation of lexical field transitions, they do not necessarily imply detrimental effects of the actual stimulation of the STN on VF performance.

The idea of negative stimulation effects on VF is based on an ‘ablational’ concept of subthalamic DBS, according to which the stimulation suppresses pathological as well as physiological STN functions [46]. However, DBS is likely to act more specifically and may normalize basic STN functions via its particular impact on abnormal signaling [47]. In this regard, DBS–just as dopaminergic treatment–has been suggested to counteract abnormal BG signaling, as shown on the level of oscillatory activity in the beta range, which is thought to unfold state-preserving functions for motor as well as cognitive processes [48]. Excessive beta-oscillations appear to prevail in most PD patients and are associated with symptoms of static motor behavior, bradykinesia and rigidity [49,50]. STN DBS diminishes this overactivity, and reduces the mentioned motor symptoms [51,52]. Since STN signaling has been proposed to mediate analogue functions for motor *and* cognitive behaviors, and low VF seems to be associated with PD [2,53], the stimulation-related switch increase and concomitant switch time reductions might be reasonably interpreted as the result of enhanced ‘antistatic’ mental drive, e.g., for disengaging from prevailing lexical concepts (clusters) towards new ones. This would also tie in with previous demonstrations of improved and faster performance in dedicated set-shifting tasks in PD patients under active compared to inactive prokinetic treatment, be it STN DBS or dopaminergic therapy [19–21]. Having said that, a correlation between the stimulation-induced switch change and motor improvement was not found in the studied patients.

In view of possible neuroanatomical underpinnings, STN DBS has specifically been proposed to reduce the excessive inhibition in PD patients mediated by the overactive hyperdirect pathway [54–56]. It may thus disinhibit deferral functions and promote go-functions [57,58] for (a review see [59])–in the present context represented by increased lexical switches. Such a background seems further in line with the correlation between the total electrical energy delivered by STN DBS and the increase in the number of switches.

### Previous findings

The general influences of STN DBS on frontal executive functions are still a matter of debate. For example, DBS-related improvements in Trail Making and Stroop tests [22] were found, in contrast to worsened associative learning [14] and response control [60,61]. As an explanation of this, STN DBS has been suggested to decrease task performances that depend on precise striatal signaling [14], but to support behaviors that benefit from the normalization of cortical hypoactivity in PD as a remote stimulation effect [62]. According to such a concept, the latter might account for the present findings. In general, DBS of the STN seems to be more susceptible to changes in cognitive scores compared to DBS of the GPi, although it has been stated that the particular influences of stimulation, trajectory, and lead placement still need to be disentangled [63].

### Limitations

The current study leaves a number of issues open. With respect to the comparison between patients and controls, strong VF differences were found, in line with previous studies demonstrating abnormally low performance in PD [2], and respective negative influence of STN DBS surgery [45]. The proper stimulation slightly counteracts these major effects by reducing switch time, but by no means normalizes VF performance. Since we only focused on this, but not on medication and surgical influences, it is speculative whether and to what extent the disease, its drug treatment, and sequels of the operation contributed to the present group distinction. Cluster analyses of VF performance in patients on and off medication and before and after DBS surgery could help to disentangle these potential factors.

With respect to the stimulation parameters, a correlation between switch increase and TEED_1sec_ in the left hemisphere was found. However, for corroborating a leading left hemispheric neuromodulation of lexical switching, further analyses with DBS in/activation per side would be needed. In view of effect dynamics, it cannot be ruled out that the switch-related changes in the present ON-OFF comparison would have been stronger, had the OFF-phase been longer than 30 minutes. Behaviorally, the stimulation-dependent changes of circumscribed VF elements suggest that neuromodulatory effects on set-shifting do not only refer to the control of largely distinct mental strategies, but also to cognitive ‘microsteps’, as reflected by lexical switching during word search. Which clinical equivalent this might have remains a topic for future investigations–given the relatively small effect size and the size of the studied group also of interest with respect to the corroboration of the current data.

### Conclusion

In sum, based on temporal cluster analysis for differentiating sub-processes of VF, the current findings support the assumption that lexical switch functions benefit from STN DBS in PD patients. Generally, this is compatible with the view that BG states shape mental processing, e.g., by regulating the flexibility to disengage from prevailing cognitive states.

## Supporting Information

S1 Table Healthy controls’ characteristics. The table shows the characteristics of subjects in the healthy control group.
(PDF)

S2 Table PD patients’ characteristics. The table shows the characteristics of subjects in the PD patient group.
(PDF)

S3 Table VF results of healthy control subjects. The table shows all results of healthy control subjects in all four VF tasks.
(PDF)

S4 Table PD patients’ VF results in the DBS ON condition. The table shows the results of PD patients in their DBS ON condition in all four VF tasks.
(PDF)

S5 Table PD patients’ VF results in the DBS OFF condition. The table shows the results of PD patients in their DBS OFF condition in all four VF tasks.
(PDF)
