# Supplementary material for: Deep Brain Stimulation of the Subthalamic Nucleus Improves Lexical Switching in Parkinsons Disease Patients
Source: PLoS One. 2016 Aug 30;11(8):e0161404. doi: 10.1371/journal.pone.0161404 (PMC5004923; doi:10.1371/journal.pone.0161404)
Supplement: S1 Table — The table shows the characteristics of subjects in the healthy control group. (PDF) [file pone.0161404.s001.pdf]

Healthy controls’ characteristics

Participant	gender male/female	age in years	education in years	handedness left/right	netPANDA
Contr1	m	63	10	R	17
Contr2	m	57	10	R	19
Contr3	f	69	10	R	18
Contr4	m	52	9	L	16
Contr5	m	71	8	L	16
Contr6	m	71	13	R	20
Contr7	f	65	13	R	23
Contr8	f	65	13	R	23
Contr9	m	66	13	R	19
Contr10	m	66	9	L	13
Contr11	m	69	10	R	21

Shown are the subject characteristics of the healthy control group.
netPANDA = Parkinson Neuropsychometric Dementia Assessment (PANDA) score without VF test items – maximum 23 points
