# Supplementary material for: Deep Brain Stimulation of the Subthalamic Nucleus Improves Lexical Switching in Parkinsons Disease Patients
Source: PLoS One. 2016 Aug 30;11(8):e0161404. doi: 10.1371/journal.pone.0161404 (PMC5004923; doi:10.1371/journal.pone.0161404)
Supplement: S2 Table — The table shows the characteristics of subjects in the PD patient group. (PDF) [file pone.0161404.s002.pdf]

PD patients' characteristics

Par	m/f	age	education	L/R	netPANDA ON	netPANDA OFF	UPDRS-III tot ON	UPDRS-III tot OFF	Hoehn Yahr	Disease Duration	LED	DBS duration ys
PD1	m	58	12	L	13	13	25	66	3	17	0.00	6
PD2	m	68	10	R	23	19	17	51	3	16	699.00	5
PD3	m	77	12	R	21	18	17	32	4	19	650.00	2
PD4	m	48	10	R	15	15	11	25	2	6	0.00	1
PD5	m	72	12	R	13	20	13	25	2	15	490.50	3
PD6	f	56	10	R	20	15	18	24	2	15	1300.00	7
PD7	f	63	8	R	16	19	40	61	3	22	400.00	2
PD8	m	70	10	R	19	17	12	23	2	11	266.00	4
PD9	m	73	8	R	21	19	13	26	2	4	1215.00	0.5
PD10	m	69	10	R	14	11	25	45	2	9	0.00	0.5
PD11	m	57	13	R	14	13	25	45	3	15	700.00	6

Shown are the subject characterists of the PD patient group.
Par = participant
m/f = gender male/female
age = age in years
education = education in years
L/R = handedness left/right
netPANDA ON = Parkinson Neuropsychometric Dementia Assessment (PANDA) score without VF test items – maximum 23 points – in the DBS ON condition
netPANDA OFF = Parkinson Neuropsychometric Dementia Assessment (PANDA) score without VF test items – maximum 23 points – in the DBS OFF condition
UPDRS-III tot ON = Unified Parkinson's Disease Rating Scale – motor score (maximum 108 points), in the DBS ON condition
UPDRS-III tot OFF = Unified Parkinson's Disease Rating Scale – motor score (maximum 108 points), in the DBS OFF condition
Hoehn Yahr = Hoehn & Yahr score
Disease duration = disease duration in years
LED = levodopa equivalent dose per day
DBS duration ys = DBS duration in years
