# Supplementary material for: Deep Brain Stimulation of the Subthalamic Nucleus Improves Lexical Switching in Parkinsons Disease Patients
Source: PLoS One. 2016 Aug 30;11(8):e0161404. doi: 10.1371/journal.pone.0161404 (PMC5004923; doi:10.1371/journal.pone.0161404)
Supplement: S3 Table — The table shows all results of healthy control subjects in all four VF tasks. (PDF) [file pone.0161404.s003.pdf]

VF results of healthy control subjects

	Phonemic non-alternating task						Phonemic alternating task						Semantic alternating task						Semantic non-alternating task					
par	N words	Sw. time	Intra-cl. time	N cl.	Cl. size	N switch	N words	Sw. time	Intra-cl. time	N cl.	Cl. size	N switch	N words	Sw. time	Intra-cl. time	N cl.	Cl. size	N switch	N words	Sw. time	Intra-cl. time	N cl.	Cl.. size	N switch
Cntr1	24	7.74	2.3	6	2.33	10	23	6.43	2.9	7	1.71	11	27	6.07	2.78	6	2.67	11	30	6.57	1.29	6	3.33	10
Cntr2	28	4.42	2.4	5	3.40	11	25	4.34	2.05	8	1.38	14	23	5.56	3.23	4	3.00	11	15	15.42	2.06	5	1.80	6
Cntr3	26	6.46	1.92	8	1.88	11	26	5.94	1.99	7	2.00	12	27	6.32	1.98	8	2.00	11	20	12.14	1.30	5	2.60	7
Cntr4	34	4.26	1.77	8	2.63	13	29	5.64	2.1	8	2.25	11	34	3.93	2	9	2.22	14	27	6.72	1.48	7	2.43	10
Cntr5	22	7.48	3.12	7	1.86	9	21	8.25	3.55	6	2.00	9	26	7.76	2.51	5	3.40	9	16	13.04	3.89	4	2.75	5
Cntr6	21	7.96	2.20	5	2.00	11	15	14.13	4.87	3	3.33	5	20	9.10	3.64	6	2.17	7	16	11.56	4.57	4	2.50	6
Cntr7	53	2.21	0.69	9	4.11	16	41	3.82	0.98	13	1.92	16	29	5.33	1.97	7	2.43	12	27	6.46	0.95	7	2.00	13
Cntr8	26	5.02	2.5	6	2.67	10	17	9.44	1.35	6	1.67	7	24	9.54	1.64	6	2.67	8	23	5.55	3.44	5	2.60	10
Cntr9	45	2.95	1.41	11	2.64	16	37	4.23	1.76	10	2.40	13	28	8.48	1.74	7	2.86	8	18	10.70	1.94	4	3.00	6
Cntr10	25	6.07	0.71	9	1.56	11	18	9.63	1.11	5	2.40	6	24	7.05	1.41	5	3.00	9	24	6.76	0.54	4	3.25	11
Cntr11	13	12.12	1.31	4	2.25	4	21	8.93	2.44	5	3.00	7	20	12.92	2.62	4	3.75	6	16	10.22	2.71	4	2.00	9

Shown are all results of the healthy control subjects in all four VF tasks.
par = participant
N words = total number of generated words
switch time = mean pause length between words belonging to separate clusters in sec.
Intra-cl. time = mean pause length between words belonging to the same cluster
N cl. = total number of produced clusters
Cl. size = mean number of clusters
N switch = total number of produced switches
