# Supplementary material for: Deep Brain Stimulation of the Subthalamic Nucleus Improves Lexical Switching in Parkinsons Disease Patients
Source: PLoS One. 2016 Aug 30;11(8):e0161404. doi: 10.1371/journal.pone.0161404 (PMC5004923; doi:10.1371/journal.pone.0161404)
Supplement: S4 Table — The table shows the results of PD patients in their DBS ON condition in all four VF tasks. (PDF) [file pone.0161404.s004.pdf]

PD patients' VF results in the DBS ON condition

	Phonemic non-alternating task						Phonemic alternating task						Semantic alternating task						Semantic non-alternating task					
par	N words	Sw. time	Intra-cl. time	N cl.	Cl. size	N switch	N words	Sw. time	Intra-cl. time	N cl.	Cl. size	N switch	N words	Sw. time	Intra-cl. time	N cl.	Cl. size	N switch	N words	Sw. time	Intra-cl. time	N cl.	Cl. size	N switch
PD1	15	9.75	3.98	4	1.75	8	13	12.15	3.89	5	1.40	6	18	9.84	3.36	4	2.75	7	11	18.64	4.70	4	1.50	5
PD2	28	6.05	2.1	7	2.57	10	24	7.57	2.29	5	2.80	10	27	7.47	1.28	5	3.20	11	25	5.52	2.14	6	2.50	10
PD3	16	9.31	3	5	1.60	8	9	26.31	5.24	3	1.67	4	15	15.52	0.6	5	1.40	8	8	32.34	4.13	3	1.67	3
PD4	34	5.52	1.47	9	2.33	13	18	9.26	4.58	4	2.75	7	23	8.01	3.23	8	1.75	9	19	10.85	2.80	4	2.75	8
PD5	22	10.79	2.2	5	3.00	7	31	6.06	2.6	8	2.88	8	29	7.03	1.80	8	2.50	9	23	12.44	1.83	6	2.67	7
PD6	20	10.17	1.70	6	2.00	8	13	14.33	6.79	4	2.25	4	17	10.86	0.57	7	1.00	10	14	9.60	0.77	3	2.00	8
PD7	19	12.17	2.94	4	3.00	7	17	9.56	3.02	6	1.33	9	21	10.07	2.66	4	3.25	8	18	13.46	3.42	3	4.00	6
PD8	12	11.89	3.65	3	1.67	7	13	19.51	4.15	2	4.50	4	21	8.87	2.92	6	2.00	9	10	18.34	4.71	3	1.67	5
PD9	20	10.42	2.98	7	1.71	8	10	23.78	5.92	3	2.00	4	22	6.66	2.93	7	1.71	10	15	22.23	3.67	3	3.67	4
PD10	4	5.66	27.99	1	2.00	2	5	95.00	3.67	2	1.50	2	16	10.60	4.86	6	1.50	7	9	13.98	4.66	3	1.67	4
PD11	20	7.37	3.14	5	2.60	7	13	11.53	5.47	4	1.75	6	15	12.00	4.89	5	1.80	6	9	10.81	4.88	2	2.50	4

Shown are the results of PD patients in their DBS ON condition in all four VF tasks.
par = participant
N words = total number of generated words
switch time = mean pause length between words belonging to separate clusters in sec.
Intra-cl. time = mean pause length between words belonging to the same cluster
N cl. = total number of produced clusters
Cl size = mean number of clusters
N switch = total number of produced switches
