# Supplementary material for: Deep Brain Stimulation of the Subthalamic Nucleus Improves Lexical Switching in Parkinsons Disease Patients
Source: PLoS One. 2016 Aug 30;11(8):e0161404. doi: 10.1371/journal.pone.0161404 (PMC5004923; doi:10.1371/journal.pone.0161404)
Supplement: S5 Table — The table shows the results of PD patients in their DBS OFF condition in all four VF tasks. (PDF) [file pone.0161404.s005.pdf]

PD patients' VF results in the DBS OFF condition

	Phonemic non-alternating task						Phonemic alternating task						Semantic alternating task						Semantic non-alternating task					
par	N words	Sw. time	Intra-cl. time	N cl.	Cl. .size	N switch	N words	Sw. time	Intra-cl. time	N cl.	Cl. size	N switch	N words	Sw. time	Intra-cl. time	N cl.	Cl. size	N switch	N words	Sw. time	Intra-cl. time	N cl.	Cl. size	N switch
PD1	12	18.07	5.68	3	2.67	4	5	60.36	8.65	1	3.00	2	13	13.04	4.03	3	2.67	5	4	4.08	7.45	2	1.00	2
PD2	22	9.20	2.57	4	3.50	8	22	8.45	2.6	7	1.86	9	24	9.20	2.49	6	2.83	7	23	9.90	1.42	8	1.75	9
PD3	13	22.56	4.09	3	3.00	4	10	21.21	7.43	3	2.00	4	12	11.12	2.23	3	2.00	6	10	41.72	4.13	3	2.33	3
PD4	29	7.24	2.06	6	3.33	9	20	9.73	3.21	7	1.71	8	22	9.29	2.39	6	2.33	8	25	5.79	1.92	7	2.14	10
PD5	27	6.90	2.23	5	4.00	7	10	15.51	3.09	4	1.50	4	20	11.13	2.32	5	2.40	8	18	9.04	3.34	6	1.67	8
PD6	22	11.76	2.66	6	2.67	6	10	19.26	6.4	4	1.25	5	19	7.97	2.45	6	1.50	10	19	12.52	1.67	4	3.25	6
PD7	26	7.68	2.3	7	2.43	9	15	13.29	4.46	5	1.80	6	24	9.62	2.54	7	2.43	7	18	7.49	4.65	3	3.33	8
PD8	12	11.98	7.71	4	1.25	7	9	15.48	6.06	2	2.50	4	17	11.55	4.93	4	3.00	5	9	60.47	7.37	2	3.50	2
PD9	9	13.69	7.08	3	1.00	6	17	8.86	4.30	5	1.80	8	19	9.32	2.30	6	2.00	7	14	12.81	3.85	4	1.75	7
PD10	9	39.23	9.58	2	3.50	2	4	83.91	3.71	2	1.00	2	17	8.44	4.38	5	1.60	9	9	16.12	9.19	2	2.50	4
PD11	9	20.25	7.17	3	2.00	3	12	13.10	5.78	5	1.40	5	20	7.66	4.64	5	2.40	8	14	6.61	1.99	5	1.40	7

Shown are the results of PD patients in their DBS OFF condition in all four VF tasks.

par = participant
N words = total number of generated words
switch time = mean pause length between words belonging to separate clusters in sec.
Intra-cl. time = mean pause length between words belonging to the same cluster

N cl. = total number of produced clusters
Cl size = mean number of clusters
N switch = total number of produced switches
